# Isolated heptadactylia: A case report of central polydactyly of the foot

**DOI:** 10.1097/MD.0000000000008324

**Published:** 2017-10-20

**Authors:** Nicolas Piette, Pierre-Yves Zambelli, Daniel N’Dele

**Affiliations:** Pediatric Orthopedics and Traumatology Unit, Lausanne University Hospital, Lausanne, Switzerland.

**Keywords:** central polydactyly, congenital foot anomaly, heptadactyly

## Abstract

**Rationale:**

Heptadactylia is a rare congenital disorder from the polydactyly family. Polydactyly is generally classified into 3 major groups: preaxial (medial ray), postaxial (lateral ray), and central polydactyly. Most common cases are related to preaxial or postaxial polydactyly. The rarity of central polydactyly can be explained in 3 ways. First, central polydactyly with duplication appearing on metatarsal is pretty uncommon. Second, the duplication appears isolated on the foot. Polydactyly is mostly associated with other physical defects or others duplications. Last, the duplication of the digital rays does not appear once but twice concerning all the digital rays and makes 7 functional toes appear. We describe this malformation with supporting iconography and radiography as well as its surgical management and functional results.

**Patient concerns:**

We analyzed an original case of isolated heptadactylia on the foot of a 14-month-old girl. The supernumerary toes made it impossible for the child to wear standard shoes and her parents were worried about this problem.

**Diagnoses:**

Clinical foot examination and radiographs revealed the presence of 7 complete rays. Every toe was composed of phalanx and metatarsal ray. There was no other congenital deformity.

**Interventions:**

Decision was made to resect the second and third rays (the two most misaligned toes in our consideration). The first stage of surgery was the ray resection and the second stage was the reconstruction of the intermetatarsal ligament to achieve a good functional and cosmetic results.

**Outcomes:**

After wound healing, the child was able to walk alone while wearing normal shoes.

**Lessons:**

We demonstrated that treatment of foot polydactyly requires careful preoperative assessment, including radiographs and photography. A good clinical evaluation of the medial polydactyly improves type-specific recognition which may enhance the accuracy of surgical treatment. Polydactyly is frequently associated with other malformations. We recommend performing a general clinical examination to exclude concomitant malformations. We recommend surgical treatment around the onset of walking.

## 1 Introduction

Heptadactylia is a rare congenital disorder belonging to the polydactyly group and causing the subject to have 7 fingers or toes. It is characterized by supernumerary digital or metatarsal toes. This condition is a prevalent birth abnormality observed in around 0.3 to 1.3 cases per 1000 newborns in the Caucasian population.^[1]^ Frequency of polydactyly varies widely among populations. The African population has a higher number observed with a 3.6 to 13.9 cases per 1000 newborns.^[2]^ No sex predilection has been identified to date.^[1]^

This disorder can be a feature of complex genetic syndromes or occur isolated. Isolated polydactyly has a normal caryotype but this does not exclude genetic aberrations.^[3,4]^

Polydactyly is generally classified into 3 major groups according to the Temtamy and McKusick classification^[4]^: preaxial (medial ray; 15%), postaxial (lateral ray; 79%), and central polydactyly (6%).^[5]^ The duplication may appear at the distal and middle phalanx or at the whole digit and metatarsal.

We describe an infant with heptadactylia. The duplication of the metatarsal without associated physical or internal organ defects was observed. In the literature, 1 case of heptadactylia is described. This case is associated with tibial aplasia.^[6]^

Surgical management of heptadactyly is challenging as there is no existing consensus or study regarding the treatment of this malformation.

## 2 Case report

**Figure 1:**
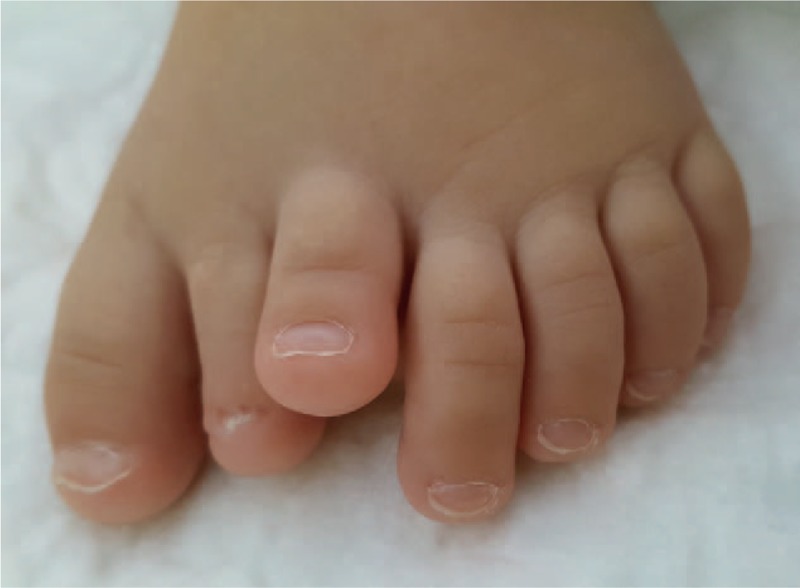
Clinical dorsal foot examination.

**Figure 2:**
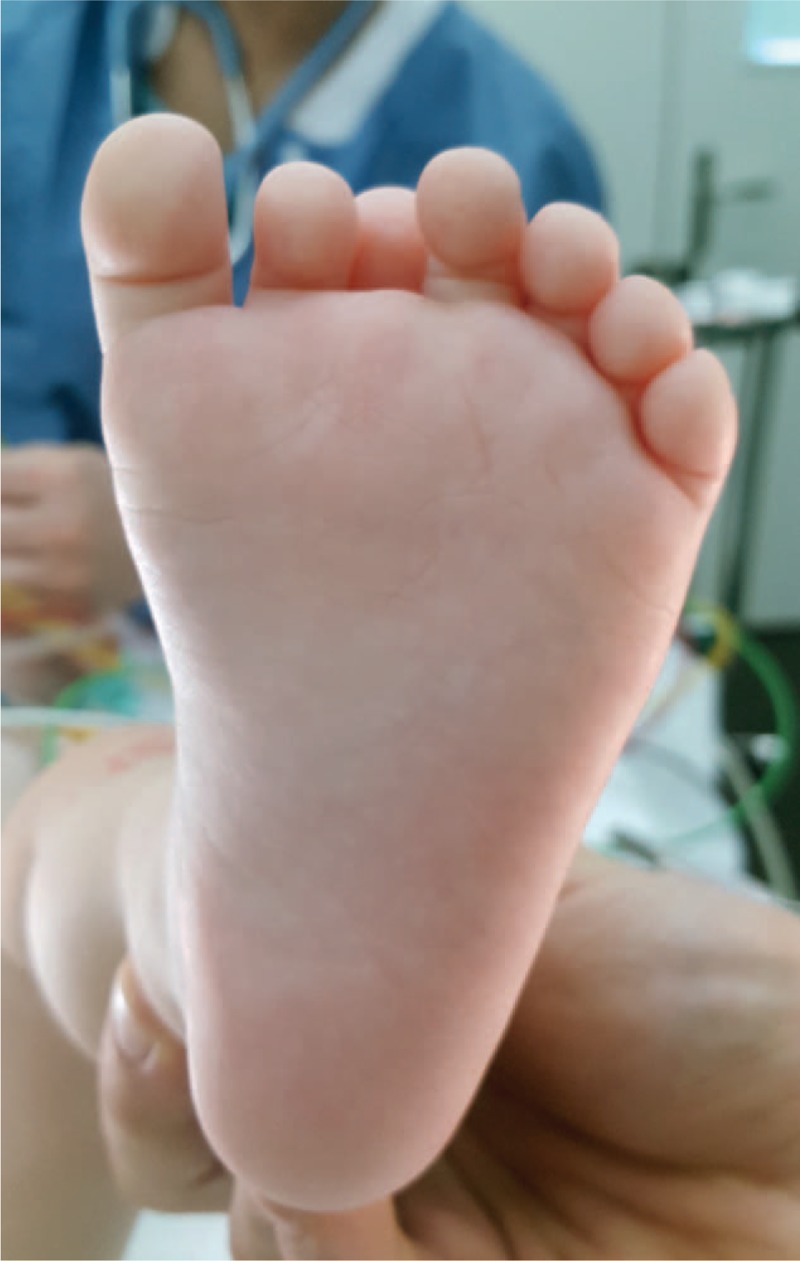
Clinical plantar foot examination.

A nonambulatory 14-month-old girl presented 7 toes on her left foot (Figs. 1 and 2). The supernumerary toes made it impossible for the child to wear standard shoes.

The girl presented a large foot with 7 toes. Heptadactylia was associated with elevation and clinodactyly of third toe and hypoplasia of the second toe. In standing position, there was ground contact of all toes except the third one which remained elevated. The metatarsal arch of the left foot measured 53 mm. The right foot had a normal appearance. The metatarsal arch measured 44 mm. We observed no other congenital deformity or malformation in this infant. No genetic study was performed.

**Figure 3:**
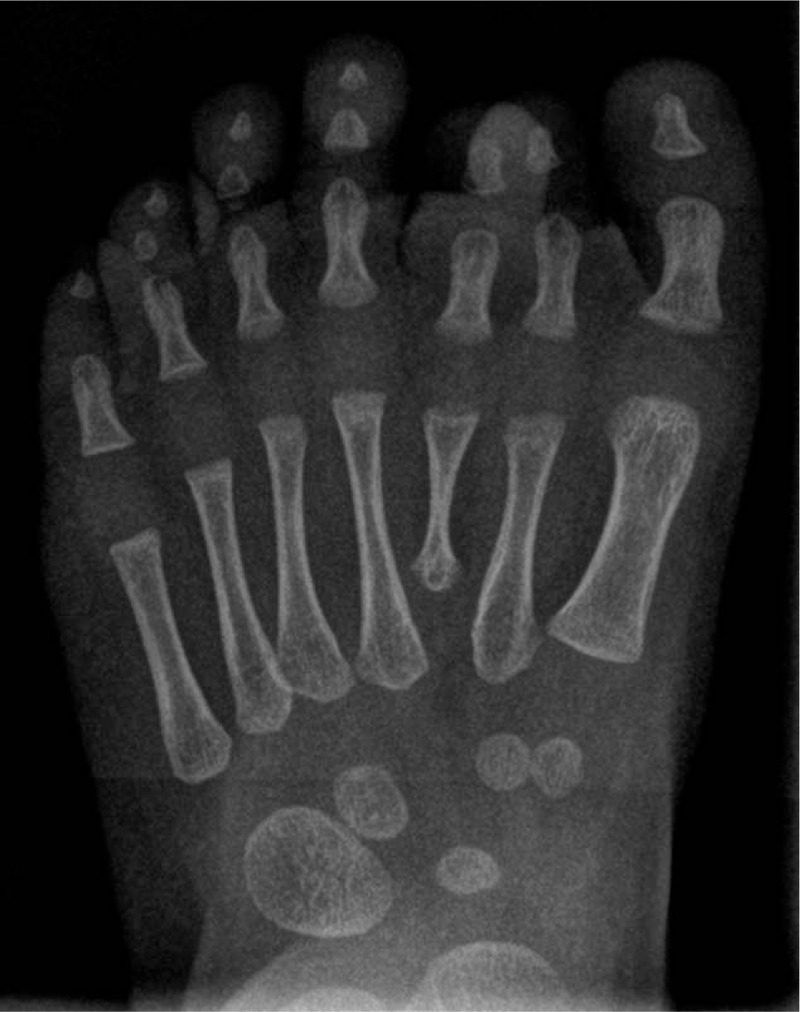
Foot radiographs revealed the presence of 7 complete rays (frontal plane).

**Figure 4:**
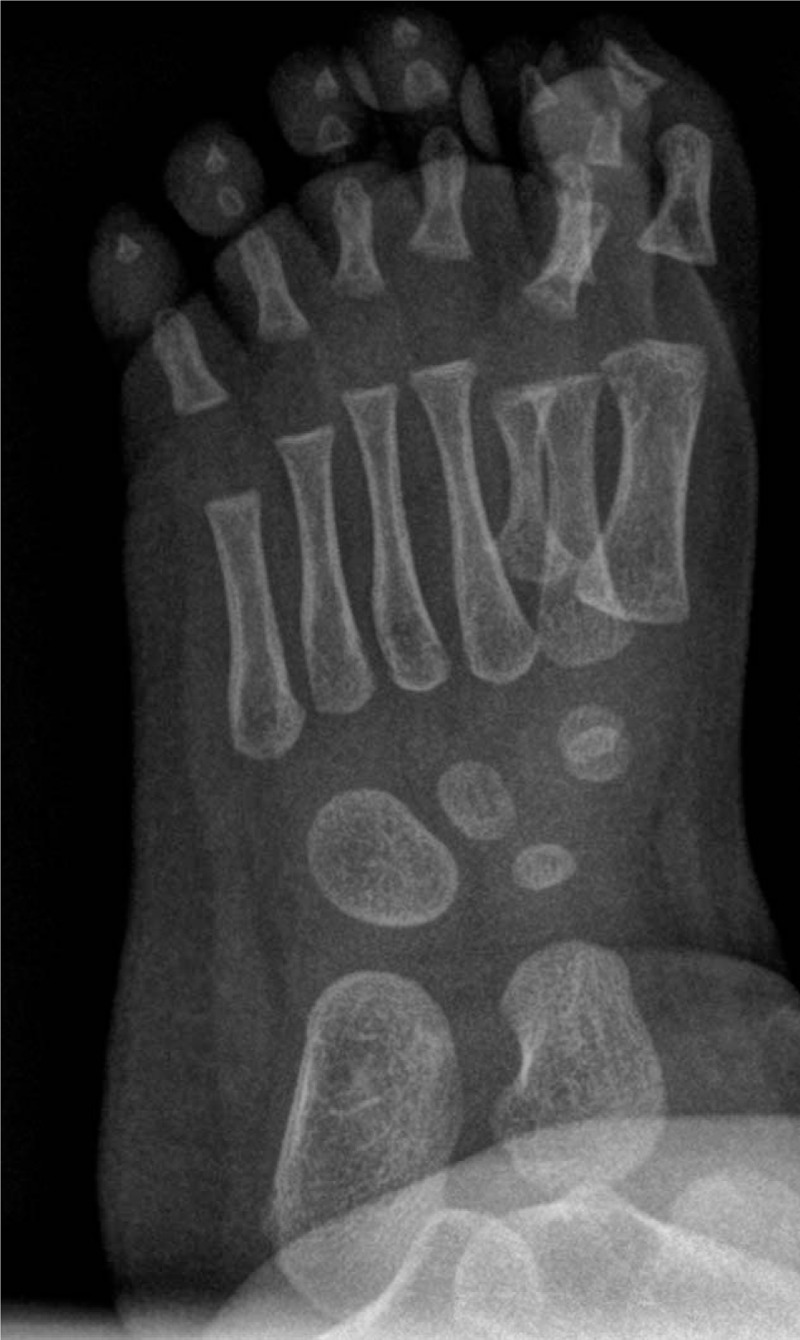
Foot radiographs revealed the presence of 7 complete rays (oblique plane).

Foot radiographies revealed the presence of 7 complete rays (Figs. 3 and 4). Every toe was composed of completely developed phalanges and metatarsal ray. There was no anomaly of tarsal bones.

**Figure 5:**
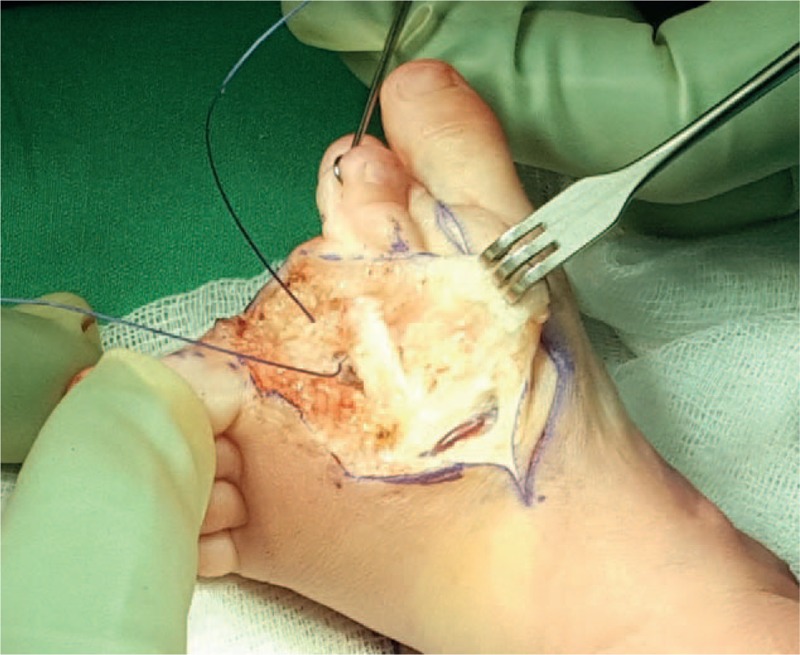
Toe excision and reconstruction of intermetatarsal ligament.

**Figure 6:**
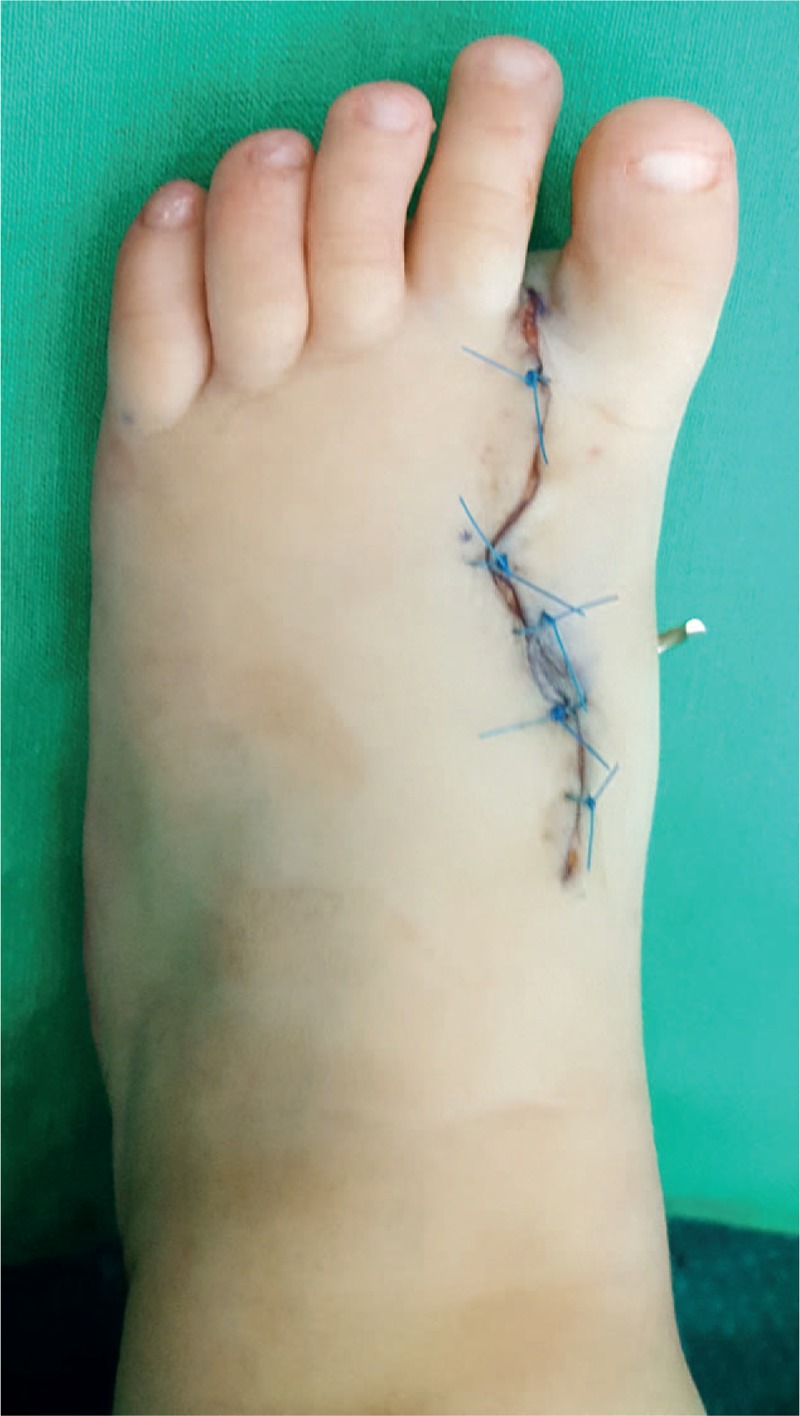
The foot just after surgery with K-wire.

We performed resection of the second and third rays; the 2 most misaligned toes in our opinion. The aim of the surgery was to provide the child with a normal functioning plantigrade narrow foot to allow the child to wear standard shoes. Our surgical approach consisted in an interdigital incision at the dorsal part of the foot. We removed the second and third rays. Dissection was carried out carefully to preserve the intermetatarsal ligament. The second stage was the reconstruction of the intermetatarsal ligament of the first and fourth metatarsals using sutures on the ligament and a K-wire to stabilize the foot (Figs. 5 and 6). After skin closure we applied a plaster cast to reduce the mobility of the reconstructed interdigital ligament. We removed the plaster and the K-wire after 4 weeks of non-weight bearing. An orthosis was then applied.

**Figure 7:**
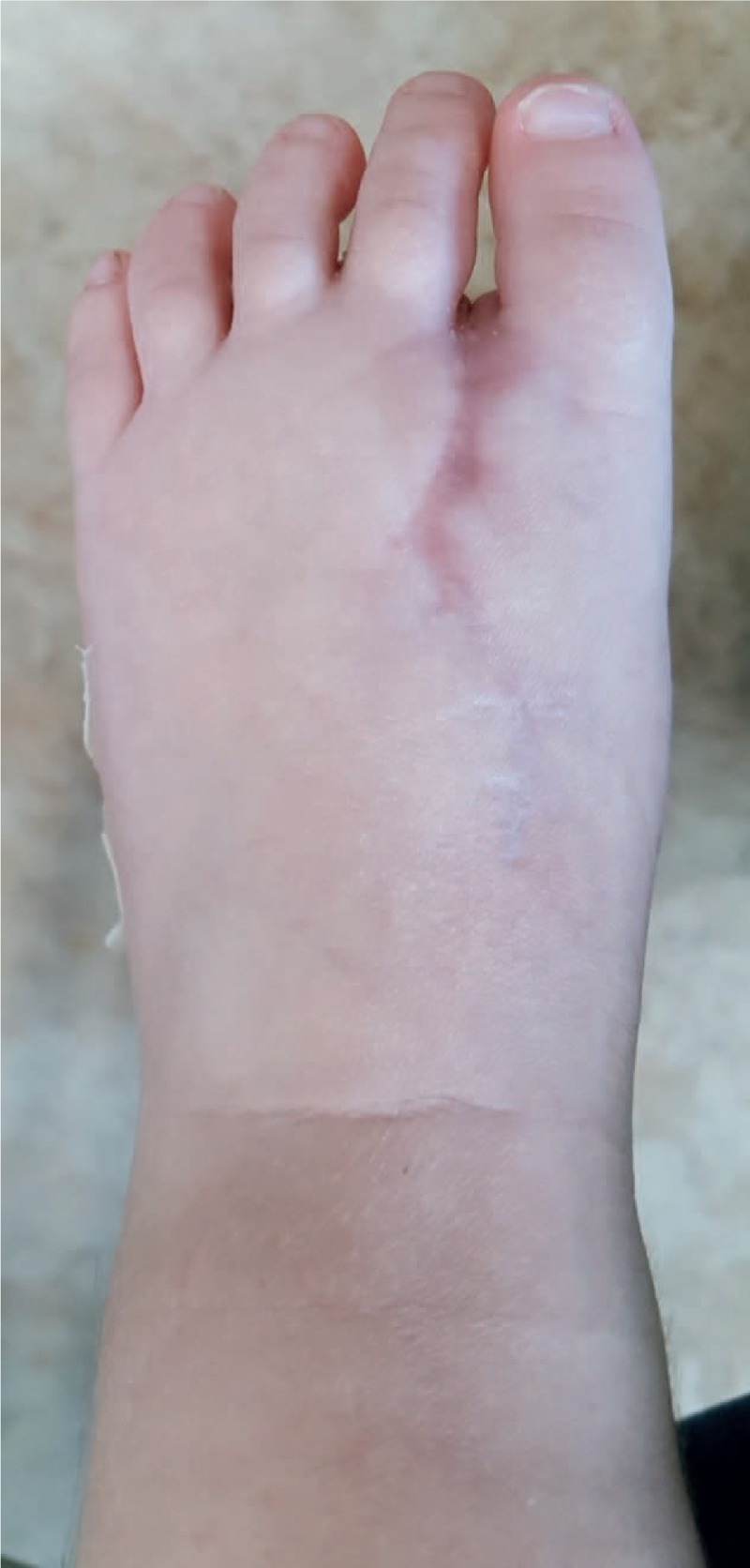
Clinical dorsal foot examination post surgery.

**Figure 8:**
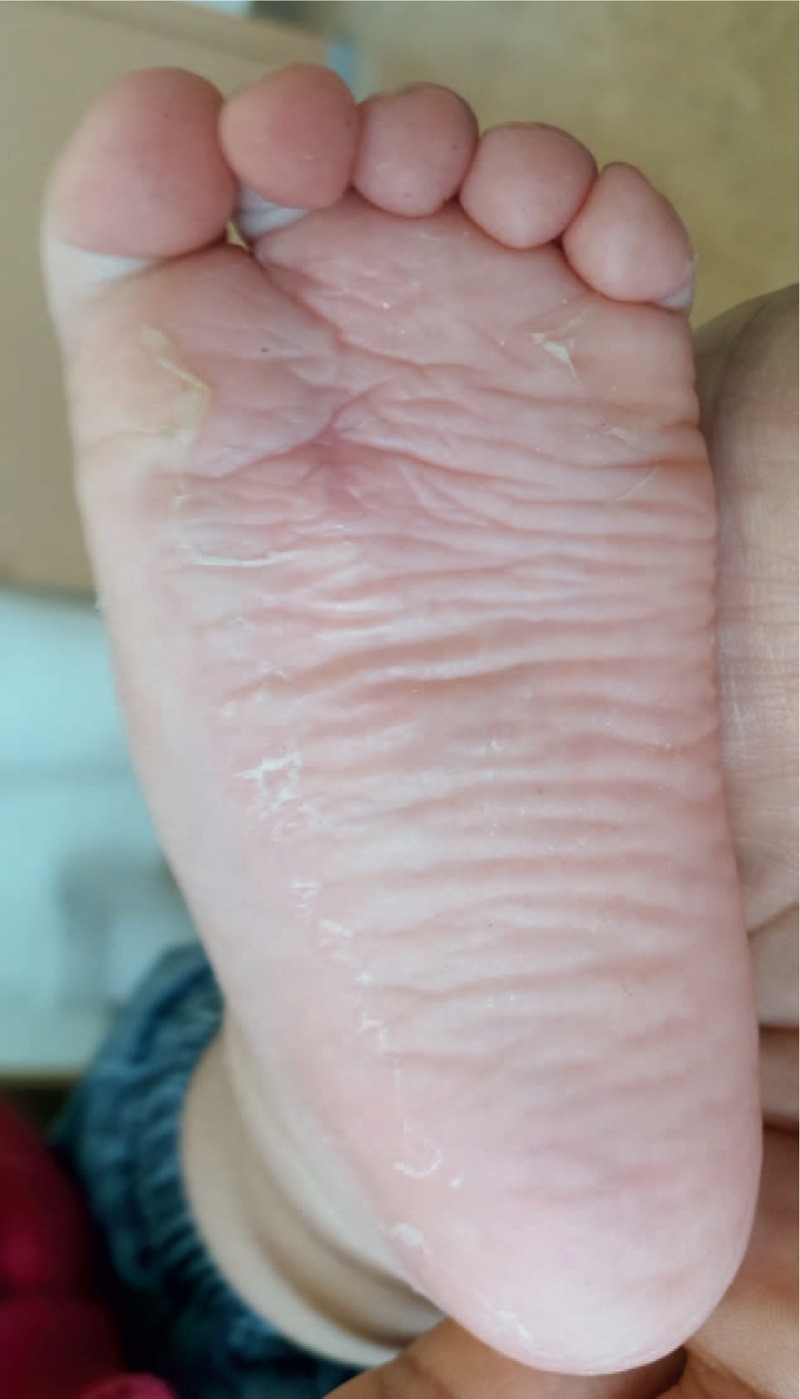
Clinical plantar foot examination post surgery.

**Figure 9:**
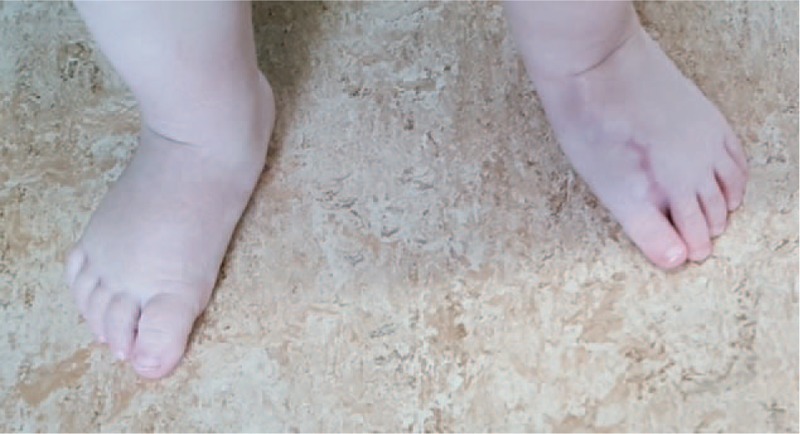
Clinical walking examination post surgery.

**Figure 10:**
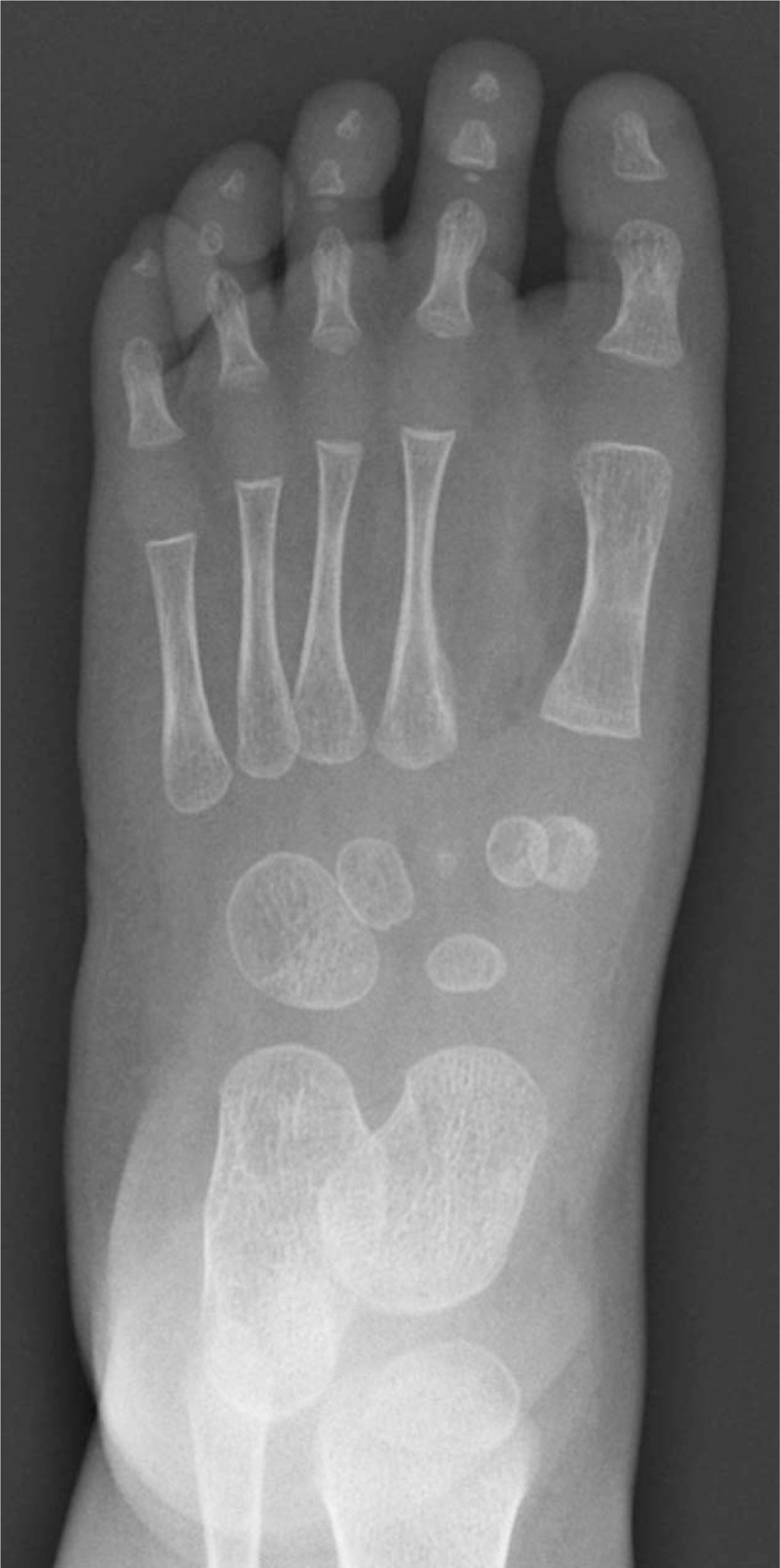
X-ray of final result (frontal plane).

**Figure 11:**
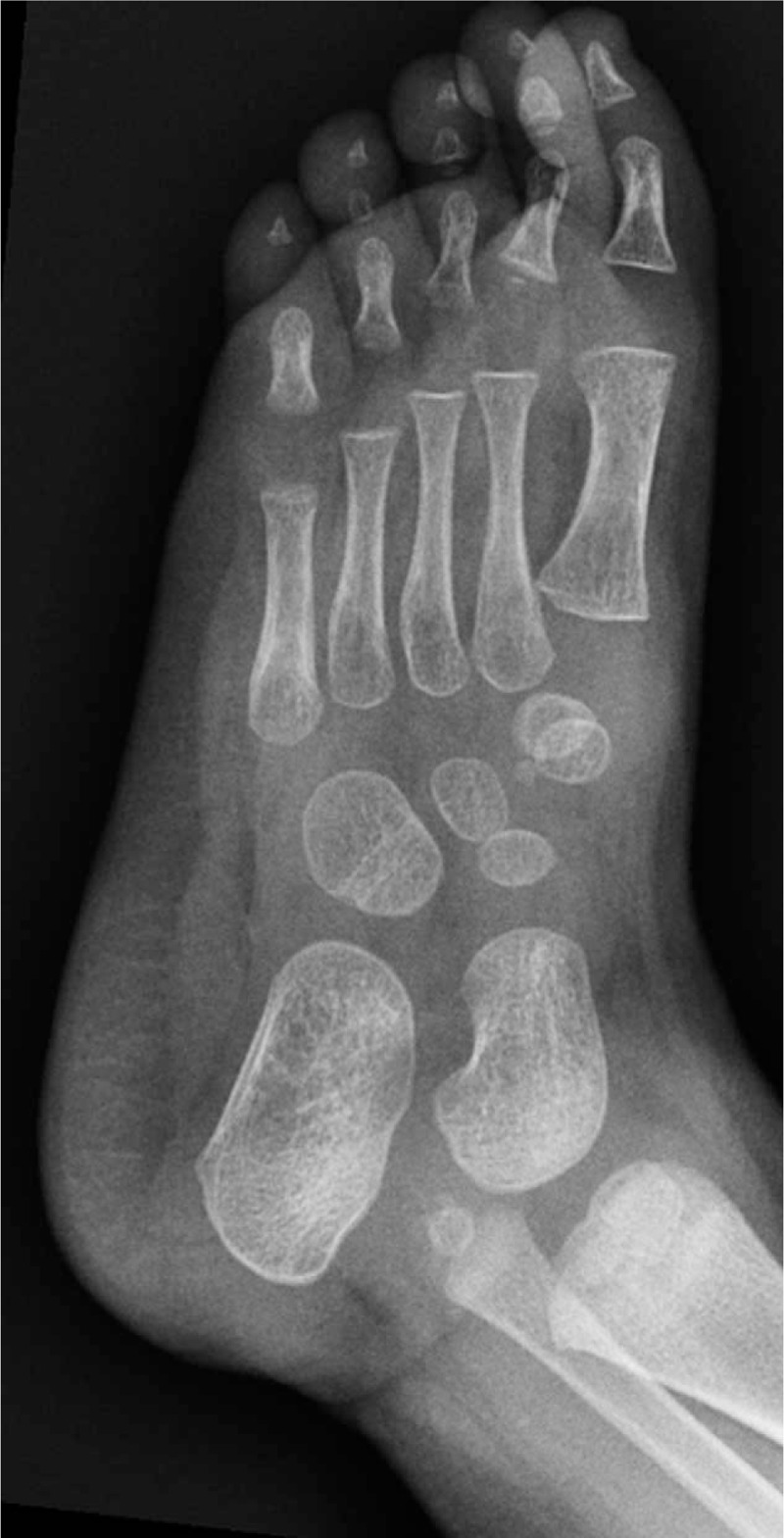
X-ray of final result (oblique plane).

The narrowness of the foot, the scar and the radiographic findings were evaluated at 3 months. The patient showed a well-healed scar (Figs. 7–9). At the same time, foot radiography was obtained which showed harmonious toes cascade alignment (Figs. 10 and 11). At this time the child was able to walk independently wearing standard shoes.

We observed no postoperative complications.

## 3 Discussion

Treatment options of polydactyly depend on the type and the underlying features.

In the literature, different surgical treatments for lateral and medial polydactyly are described. Publications about central polydactyly are very rare. Currently, there is no standard protocol on the treatment of heptadactylia.

In central polydactyly, the metatarsal extension is an important component of the malformation and can influence the surgical treatment approach of the duplicated metatarsal bone. The foot's metatarsal bones play a significant role in sustaining the transverse arch of the foot. Therefore, we must understand the extent to which the metatarsal duplication of the polydactyly has to be treated.^[7,8]^

The clinical signs and symptoms of polydactyly are diverse and include spontaneous pain, excruciating walking tenderness, ill-fitting shoe concerns, cosmetic problems, and psychosocial difficulties.

We recommend delaying surgery and resisting the urge to have surgery on newborns for esthetical reasons. In the literature, polydactyly of the foot is usually treated during infancy after onset of walking. Many authors advocated delaying surgery until skeletal development (ossification) has occurred within the affected rays so that accurate anatomic assessment is possible.^[9]^ Our current practice is to delay surgery until 1 year of age. One reason is at this age, there is a better definition of the bony anatomy and the duplicated structures on the radiographic pictures are defined better. Second, at this age, the child starts to walk and we can better evaluate whether polydactyly causes gait problems.

Surgery for central ray duplication should be performed very cautiously as it is very difficult to obtain a narrow foot.^[7,10]^ The success of treatment depends on the reconstruction of the intermetatarsal ligament after excision of the metatarsal and the toe to preserve the transverse arch. We also recommend applying a postoperative cast and taping to protect the intermetatarsal ligament reconstruction and to prevent deformities like collapse of the foot.

## 4 Conclusion

We report a rare case of unilateral heptadactylia involving central rays of the foot in an infant with no other physical abnormalities. We recommend performing a general clinical examination to exclude concomitant malformations or organ defect as polydactyly is frequently associated with other malformations.

Treatment of central polydactyly of the foot requires careful preoperative assessment, including radiographs and photographs as no standard protocol on treatment of polydactyly exists at present. Case-specific treatment should be applied and tailored to meet the individual needs. Metatarsal bones play a significant role in establishing the transverse arch of the foot. It is of utmost the importance to reconstruct the intermetatarsal ligament to restore the transverse arch and obtain a narrow foot.

## 5 Consent

Written informed consent was obtained from the patient's legal guardian concerning publication of this manuscript and accompanying images.
